# Effects and treatment methods of acupuncture and herbal medicine for premenstrual syndrome/premenstrual dysphoric disorder: systematic review

**DOI:** 10.1186/1472-6882-14-11

**Published:** 2014-01-10

**Authors:** Su Hee Jang, Dong Il Kim, Min-Sun Choi

**Affiliations:** 1 Department of Korean Gynecology, College of Korean Medicine, Dongguk University, Seoul, South Korea; 2 Department of Acupuncture and Moxibustion, Nasaret Oriental Medical Hospital, Inchon, South Korea

**Keywords:** Premenstrual syndrome, Premenstrual dysphoric disorder, Acupuncture, Herbal medicine, TCM, CAM, PMS, PMDD

## Abstract

**Background:**

During their reproductive years about 10% of women experience some kind of symptoms before menstruation (PMS) in a degree that affects their quality of life (QOL). Acupuncture and herbal medicine has been a recent favorable therapeutic approach. Thus we aimed to review the effects of acupuncture and herbal medicine in the past decade as a preceding research in order to further investigate the most effective Korean Medicine treatment for PMS/PMDD.

**Methods:**

A systematic literature search was conducted using electronic databases on studies published between 2002 and 2012. Our review included randomized controlled clinical trials (RCTs) of acupuncture and herbal medicine for PMS/PMDD. Interventions include acupuncture or herbal medicine. Clinical information including statistical tests was extracted from the articles and summarized in tabular form or in the text. Study outcomes were presented as the rate of improvement (%) and/or end-of-treatment scores.

**Results:**

The search yielded 19 studies. In screening the RCTs, 8 studies in acupuncture and 11 studies in herbal medicine that matched the criteria were identified. Different acupuncture techniques including traditional acupuncture, hand acupuncture and moxibustion, and traditional acupuncture technique with auricular points, have been selected for analysis. In herbal medicine, studies on Vitex Agnus castus, Hypericum perforatum, Xiao yao san, Elsholtzia splendens, Cirsium japonicum, and Gingko biloba L. were identified. Experimental groups with Acupuncture and herbal medicine treatment (all herbal medicine except Cirsium japonicum) had significantly improved results regarding PMS/PMDD.

**Conclusions:**

Limited evidence supports the efficacy of alternative medicinal interventions such as acupuncture and herbal medicine in controlling premenstrual syndrome and premenstrual dysphoric disorder. Acupuncture and herbal medicine treatments for premenstrual syndrome and premenstrual dysphoric disorder showed a 50% or better reduction of symptoms compared to the initial state. In both acupuncture and herbal medical interventions, there have been no serious adverse events reported, proving the safety of the interventions while most of the interventions provided over 50% relief of symptoms associated with PMS/PMDD. Stricter diagnostic criteria may have excluded many participants from some studies. Also, depending on the severity of symptoms, the rate of improvement in the outcomes of the studies may have greatly differed.

## Background

Premenstrual syndrome (PMS) is a psychological, behavioral, and physical symptom that occurs during the late luteal phase of the menstrual cycle and disappears by the onset of menstruation [1]. As much as 25% of menstruating women report moderate-to-severe premenstrual symptoms. Approximately 5% report severe symptoms [2].

In a telephone survey done in the U.S., 80% of women preferred non-pharmacological interventions, such as vitamins and supplements or alternative methods of treatments. The suggested etiology of PMS includes abnormal neurotransmitter responses to normal ovarian functions, hormonal imbalances, sodium retention, or nutritional deficiencies [3]. Pharmacologic treatments have included antidepressants (selective serotonin reuptake inhibitors, SSRIs) and other psychotropic agents, diuretics, progesterone, GnRh agonists, hormonal therapy such as estrogen therapy, combined oral contraceptives, pyridoxine, ethinyl estradiol and drospirenone, and synthetic androgen and gonadotropin inhibitors [4]. However, more women were found to prefer non-pharmaceutical approaches including dietary changes, exercise, cognitive behavioral therapy, and complementary and alternative medicine [5]. As for the most recent systematic review and meta-analysis of complementary and alternative medicine on PMS and PMDD, Kim *et al*. [6] in 2011 showed favorable results.

The specific objectives of this review were; (1) to identify types of acupuncture methods and herbal medicine used in treating PMS/PMDD; (2) to identify the efficacy of the interventions; (3) and to compare the mean differences for each symptom of the syndrome/disorder.

## Methods

### Search strategy

**Figure 1:**
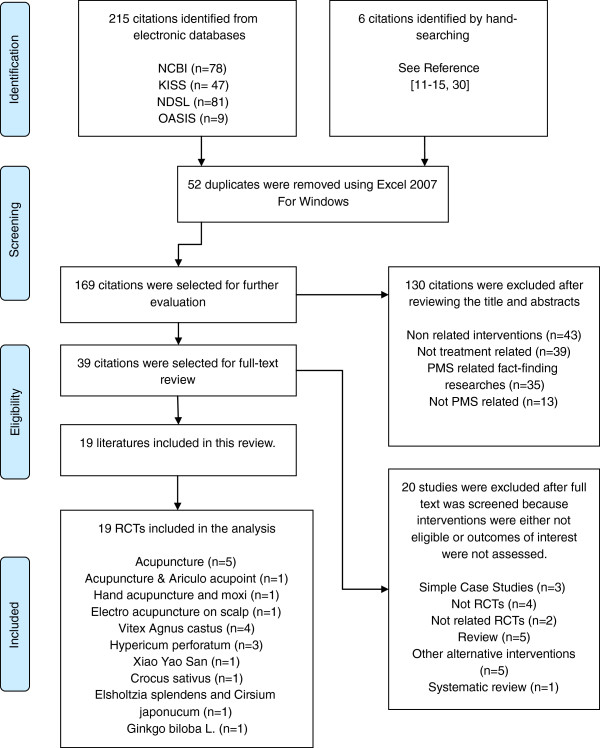
**Flow chart.** Search and selection criteria conducted in accordance with PRISMA statement criteria. (Preferred Reporting Items for Systematic Reviews and Meta-Analyses).

Under the guidelines of Preferred Reporting Items for Systematic Reviews and Meta-Analyses (PRISMA) [7] a systematic literature search was done by two authors on studies that were published between the time frame of January 2002 to September 2012 in four electronic databases: Pubmed, KISS (Korean-studies information service system), NDSL (national discovery for science leaders), OASIS (Oriental Medicine Advanced Searching Integrated System) (Figure 1) (Additional file 1). Six articles were manually searched. The following search terms were used: premenstrual syndrome acupuncture, premenstrual syndrome alternative medicine, premenstrual syndrome herbal medicine, premenstrual syndrome CAM, premenstrual dysphoric disorder acupuncture, premenstrual dysphoric disorder alternative medicine, premenstrual dysphoric disorder herbal medicine, premenstrual dysphoric disorder CAM for Pubmed. Search terms Premenstrual syndrome (Korean and English) and premenstrual dysphoric disorder (Korean and English) were used for the remaining search. The search was conducted to identify studies reporting acupuncture and herbal medical treatments of premenstrual syndrome or premenstrual dysphoric disorder. The literature search process is illustrated in Figure 1. Data were recorded and assessed using Excel 2007 FOR WINDOWS version.

### Inclusion and exclusion criteria

Included studies met the following criteria. (1) Randomized controlled clinical trials (RCTs), (2) participants of the study were diagnosed for PMS or PMDD, (3) the study compared acupuncture with control groups or herbal medicine including multi-component herbal formulation with placebo or pharmaceutical medicine, (4) the study used outcome measures to show the changes in PMS symptoms before and after the treatment. Other interventions such as Qi therapy, yoga, exercises, homeopathy and pharmaceutical medicine were excluded. Case reports, theoretical treatment methods were excluded from the study. Literatures published before January 2002 and after September 2012 were excluded.

### Data extraction

Study selection, data extraction and risk of bias assessment, and quality assessment were performed independently by the first author under provision of the second author. The condition, trial sample size, study duration, herbal extract and dosage regimen, intervention methods, and outcome measures were extracted from the selected literatures. The multi-components Xiao Yao San and Dan Zhi Xiao Yao San were herbal granules, commonly accepted substances with no major known side effects published and are approved by the Therapeutic Good Administration (TGA), Australia [8]. The authors of the research have changed their email addresses and because they were no longer listed at the facility mentioned in the article, the exact formulation could not be verified. The general ingredients of Xiao Yao San are Chae Hu (Radix Burpleuri) 75 mg, Bai Zhu (Rhizome Atractylodis macrocephalae) 75 mg, Fu Ling (Poria) 75 mg, Dang Gui (Radix Angelicae sinensis) 75 mg, Bae Shao (Radix Paeoniae alba) 75 mg, Shen Jiang (uncooked Rhizoma Zingiberis) 50 mg, Bo He (herba Menthae haplocalycis) 50 mg, Zhi Gan Cao (honey fried Radix Glycyrrhizae uralensis) 25 mg, and the general ingredients of Dan Zhi Xiao Yao San are Mu Dan Pi (Cortex Moutan) 68.2 mg, Zhi Zi (Fructus Gardneiae) 68.2 mg, Chai Hu (Radix Bupleuri) 68.2 mg, Dang Gui (Radix Angelicae sinensis) 68.2 mg, Bai Shao (Radix Paeoniae alba) 68.2 mg, Bai Zhu (Rhizoma Atractylodis macrocephalae) 68.2 mg, Fu Ling (Poria) 68.2 mg, Gan Cao (Radix Glycyrrhizae) 22.6 mg).

### Calculation for reevaluation

The outcome was reevaluated using the following valuation: Significant result (%) = (baseline score-post treatment score)/baseline score) x100. Further evaluation across studies on the efficacy of treatments based on reevaluated scores by symptoms was additionally analyzed (see Overall symptoms section).

## Results

### Data search

Through the database search, 221 literatures were identified with the aforementioned search words. After the screening process and an evaluation of the eligibility of the articles, 19 articles were identified for the final review and analysis. The articles were reviewed on the utilized treatment methods. 19 articles were randomized double or single-blind placebo-controlled studies within the past decade, designed to evaluate the efficacy of acupuncture or herbal medicine treatments for PMS/PMDD. In total, 8 acupuncture treatments and 11 herbal medical treatments were found and evaluated (see Acupuncture and Herbal Intervention). Acupuncture treatments included general acupuncture points, manipulated techniques of acupuncture, and hand acupuncture [9-16]. Herbal medical interventions included the following formulae: Xiao yao san (or Dan Zhi Xiao yao san) [8] and herbal medicine included Vitex Agnus castus, Hypericum perforatum, Crocus sativus, Elsholtzia splendens, Cirsium japonicum, and Ginkgo biloba L. [17-26].

### Summary measures

A screening questionnaire for the assessment of PMS or PMDD was limited to the following tools: DSM-IV PMDD, and DSR. In monitoring symptoms and scoring the outcome measures the tools varied but were limited to the following: Menstrual Distress Questionnaire (MDQ), Menstrual Symptoms Severity List (MSSL), Premenstrual Syndrome Diary (PMSD), Daily Symptom Rating (DSR), The Premenstrual Tension Syndrome Self-Rating Scale (PMTS), and Premenstrual Assessment Form (PAF). The percentage of reduction was defined as the difference in symptom scores between the final score after treatment and symptom score at baseline. The efficacy variable was the reduction percentage of symptom scored documented in the assessment tools listed above. The efficacy variable was the percentage of symptom scores reduced that were documented in the assessment tools listed above.

### Risk of bias across studies

The risk of bias in the studies was variable along studies. For adequate sequence generation, two studies used randomized block designs [6,8], one study used a computer-generated random number sequence to allocate patients to the treatment and control groups [16]. Thirteen studies had insufficient reports on how their random numbers were generated [9-15,18-23] (Table 1).

**Table 1:** Risk of bias of included RCTs*

**Study**	**Random sequence generation**	**Allocation concealment**	**Patient blinding**	**Assessor blinding**	**Incomplete outcome data**	**Selective outcome reporting**
Chou (2008) [8]	L	U	U	U	L	L
Kim (2005) [9]	U	U	U	U	H	H
Habek (2002) [10]	U	U	U	U	U	U
Shin (2009) [11]	U	U	H	H	U	U
Xu (2006) [12]	U	U	U	U	U	U
Xu (2006) [13]	U	U	U	U	U	U
Guo (2004) [14]	U	U	U	U	U	U
Hong (2002) [15]	U	U	U	U	U	U
Yu (2006) [16]	L	L	U	U	U	U
Ma (2010) [17]	L	L	U	U	U	U
He (2009) [18]	U	U	U	U	U	U
Atmaca (2003) [19]	U	U	L	L	U	L
Zamani (2012) [20]	U	L	L	L	L	L
Canning (2010) [21]	U	L	L	L	U	L
Hicks (2004) [22]	U	L	L	L	U	L
Masumeh (2010) [23]	U	U	U	U	U	U
Agha-Hosseini (2008) [24]	L	L	L	L	L	L

*Domains of quality assessment based on the Cochrane tools for assessing risk of bias.

Abbreviations; *L*low risk of bias, *H* high risk of bias, *U* unclear (uncertain risk of bias).

In allocation concealment, two studies adequately concealed group assignments by adopting central randomization [16,17]. One study used medications identical in appearance labeled A and B while an identification number was noted in a protocol to allow subsequent identification and statistical analysis after the completion of the study [20]. In one study women received medication in form of a tablet after being randomly assigned in a 1:1 ratio using a computer-generated code [24]. In the remaining studies allocation was not reported or unclear (Table 1).

Blinding was evaluated separately for patients and outcome assessors. Most trials had insufficient information. For outcome assessor blinding, most studies received ratings of ‘unclear’ because of poor reporting or the self-reporting nature of the outcome measures used. One study had the patients and raters blind to drug assignment [19]. One study had the identification number in a protocol while the information on the placebo and the active substance was made available to the investigators and volunteers only after the completion of the study and after the statistical analysis was performed [20]. One study had supplies packaged in plain boxes labeled with codes and study cycle numbers [21]. One study had all tablets coated to make them look identical and were supplied in plaster packs marked with the days of the week to aid compliance [22]. One study had the assignments kept in sealed, opaque envelopes until the point of data analysis. The randomization and allocation process was performed by the principle investigator of the trial who was not involved in the process of treatment and measurement [24] (Table 1).

### Acupuncture interventions

Eight studies and 9 different interventions were identified. Acupuncture treatment sessions ranged from 2 to 13 sessions and treatment periods varied from both luteal and follicular phases (L/FP) [9,11,15] to only the luteal phase (LP) [10,12-14,16]. Studies comprised of Korean acupuncture technique [9], TCM method with auriculopoint Shenmen added [10], Korean hand acupuncture and moxibustion technique [11]. On a study done in Korean acupuncture technique, points SP6, CV6 were mainly used [9]. Physical symptoms such as headache, cramps, backache, cold sweats, hot flashes, breast pain, skin disorders, swelling of hands and feet, sensitivity to cold, abdominal pain and bulging improved as much as 50.5% [9-11]. Psychological symptoms also improved, but there was no significant difference when compared with the control group [9-11]. Acupuncture treatment using SP6 CV6 as the main points resulted in the change of a MSSL score of 16.78 to 7.56 by the end of the session [9]. Treatment using DU20, LI4, H3, REN3,4,6, PE6, GB34, UB23 had a 77.8% reduction at the end of the trial [10]. Hand acupuncture and moxibustion treatments starting with an MSSL score of 20.63 and 20.65 at the initial point reduced them to 3.94 and 3.40 at the end of the session [11]. Back-shu points and Point-thought-point techniques, electroacupuncture on scalp, treatment using BL17,18,20,23 and GV20, Ex-HN2,3 all had better outcomes than the control group [12-16]. The outcome of the rest of the acupuncture interventions are listed in Table 2.

**Table 2:** Therapeutic effect of acupuncture on premenstrual syndrome

**#**	**Acupuncture points**	**Frequency**	**Sample**	**2 cycles of pre-rating**	**Baseline**	**Outcome (end-of-Tx score)**	**Control**	**Adverse**	**P-value**
		**Tx sessions**	**Size**				**(no of CG)**	**Events**	
1	SP6 CV6 + LR3, LR2, SP10, LI4 or + ST36^8^	13 @ L/FP	10	Not reported	MSSL	7.56 ± 2.36	SI 5	None reported	P < 0.05
		2/wk, 8 wks (2 cycles)			16.78 ± 4.30		ST 40		
							(10)		
2	DU20 LI4 H3 REN3,4,6 PE6 GB34 UB23, Auriculoacu-point Shenmen^9^	2 ~ 4 @ LP	18	Not reported	Diagnosed as PMS	77.8% reduction	Sham acupuncture	One subcutaneous abdominal hematoma	p < 0.008
		(1 cycle)					(17)		
3	Hand acupuncture therapy^10^	10 @ L/FP	7	Not reported	MSSL	3.94 ± 1.66	No treatment received	No serious AE observed	p < 0.001
	A5,A6,A8,A12,A16,A18,N18,F6	3/wk, 4 wks			20.63 ± 10.32		(10)		
		(1 cycle)							
4	Hand moxibustion therapy^10^	10 @ L/FP	8	Not reported	MSSL	3.40 ± 1.78	No treatment received	None reported	p < 0.001
	A5,A6,A8,A12,A16,A18,N18,F6	3/wk, 4 wks			20.65 ± 6.12		(10)		
		(1 cycle)							
5	Back-Shu points^11^	30 @ LP	20	Not reported	Met Chinese standards for diagnosis for PMS	Better than CG	Standard acupuncture	None reported	p < 0.05
	BL15,17,18,20,21,23	7/wk				score n/a	(20)		
		(3 cycles)							
6	Point-through-point^12^	30 @ LP	30	Not reported	Diagnosed for PMS by OB/GYN textbook	Better than CG	Medication - progestin (medroxyprogesterone, 6 mg daily) (30)	None reported	p < 0.05
	GV3 ~ 8 BL18 ~ 23 BL47 ~ 52	7/wk				score n/a			
		(3 cycles)							
7	BL17,18,20,23 GV20 CV4,17 SP6 PC6 LR3^13^	30 @ LP	31	Not reported	Diagnosed as DSM-IV-TR	Better than CG	Medication - medroxy-progesterone 4 mg, diazepam 2.5 mg twice daily (31)	None reported	p < 0.05
		7/wk				score n/a			
		(3 cycles)							
8	Electroacupuncture on scalp^14^	30 @ L/FP	35	Not reported	Diagnosed as PMS by OB/GYN textbook	Better than CG	Medication - medroxy-progesterone 4 mg, diazepam 2.5 mg twice daily (35)	None reported	p < 0.05
	MS1,5 + MS2,3,4	3/wk				score n/a			
		(3 cycles)							
9	GV20 Ex-HN3,5 SP6,10 + LR3 CV17 LR14 Ex-CA1 CV4 SP9 ST36 CV6 PC6 HT7 BL23 GV4 KI3^15^	21 @ LP	30	Not reported	Met ICD-10 criteria	Better than CG	Sham acupuncture	Two hypo menorrhea during 2^nd^ cycle	p < 0.05
		3 ~ 4/wk				score n/a	Selection of points N/A		
		(3 cycles)					(33)		

Literatures yield 9 studies as interventions. It comprises of acupuncture points and technique, treatment sessions marking the period of the session (either at luteal phase (LP) or at both LP and follicular phase as L/FP, Duration of the session as in weeks and by menstrual cycles, Baseline score and the outcome score, the control type, and p-value.

*n/a, not available; NS, not significantly different between groups; CG, control group.

### Herbal interventions

Eleven studies and 7 different interventions were identified. The duration of herbal medical treatments ranged from one menstrual cycle to six menstrual cycles with herbal medication taken between once to three times daily or during the luteal phase (LP) only [8,17-26]. Studies comprised of herbal medicine such as Vitex Agnus castus (4 studies) [17-20], Hypericum perforatum (3 studies) [21-23], Xiao yao san (and Dan Zhi Xiao yao san) [8], Crocus sativus [24], Elsholtzia splendens [25], Cirsium japonicum [25], and Ginkgo biloba L. [26], which were in liquid form, powder from, or tablet form. Study of Vitex Agnus castus by Ma [17] is an analysis of a sub-population of study by He [18]. VAC BNO1095 (40 mg/day, 70% extract Agnucaston^®^) was superior to placebo over 3 cycles for total PMS symptoms measured on the PMTS (p <0.001), PMSD scales (p < 0.05), and clinical efficacy rates (p <0.001). In all studies on Vitex Agnus castus, psychological and physical symptoms showed more than 50% improvement over control groups [17-20]. However, on the study done with Fluoxetine as a comparative drug, there was no significant difference between the two groups except that in the Fluoxetine group, there were two adverse events of sexual dysfunction [19]. Dosage ranged from 20 to 40 mg daily. The outcomes of the rest of the herbal intervention are listed in Table 3. One study on Vitex agnus castus is the analysis of a sub-population of a systematic review on clinical trials [19,27].

**Table 3:** The effect of herbal medicine for premenstrual syndrome

**#**	**Intervention**	**Frequency**	**Sample**	**2 cycles of pre-rating**	**Initial state M**	**Outcome**	**Control**	**Adverse**	**P-value**
	**(dosage/day) -form**	**(Tx Duration)**	**Size**			**(Improved rate or end-of-Tx score)**	**(no of CG)**	**Events**	
10	Vitex Agnus castus^17**^ (VAC, BNO 1095) 40 mg -Tablet	1/day	33	Confirmed	PMSD sum score 29.38 ± 7.63 (p = 0.752)	PMSD sum score 14.66 ± 0.52	Placebo	No notable AE observed	=0.0001
		(3 cycles)					(34)		
11	Vitex Agnus castus^18^ (VAC, BNO 1095, 4.0 mg of dried ethanolic (70%)) 40 mg -Tablet	2/day	101	Confirmed	PMSD 29.13 ± 7.88 (p = 0.4017)	PMSD 6.41 ± 7.94	Placebo	No serious AE observed	<0.05
		(3 cycles)			PMTS 26.17 ± 4.79 (p = 0.1649)	PMTS 9.92 ± 9.01	(101)		
12	Vitex Agnus castus extract^19**^ (AC extract)	1/day	19	Confirmed	DSR 171.758.1 (p > 0.05) HAM-D 15.24.7 (p > 0.05) CGI-SI 4.11.4 (p > 0.05)	DSR 82.849.5	Fluoxetine	No serious AE observed from TG	>0.1
						HAM-D 7.64.3	(19)		
	20-40 mg –Tablet	(2 cycles)				CGI-I 1.20.7 five symptoms diminished 50% or more		2CG: Sexual dysfunction	
13	Vitex Agnus castus^20^ (Vitex agnus extract)	1/day	62	Confirmed	DSR 30% higher score @ LP	Better than CG	Placebo	No serious AE observed	<0.0001
		@ LP					(66)		
	40 drops (4.5 mg) -Liquid	(6 cycles)							
14	Hypericum Perforatum^21^	2/day	17	Confirmed	DSR score in LP 12.6	DSR score 5.80 (F [1,30] = 4.82; p = 0.04; partial Z2 = 0.14)	Placebo	No serious AE observed	>0.05
	(Li 160 (80% methanolic dry extract, 0.18% hypericin, 3.38% hyperforin) 900 mg -Tablet	(2 cycles)					(15)		
15	Hypericum Perforatum^22^ (St. John’s wart extract, 300 mg of extract, 900 ug of hypericin) 1800ug hypericin (600 mg) -Tablet	2/day	64	Confirmed	MD score 326.33§	MD score 230.28 (p ≤ 0.007)	Placebo	No serious AE observed	<0.007
							(61)		
		(2 cycles)							
16	Hypericum Perforatum^23^	2/day	85	Confirmed	DSR 149.07	DSR 86.13	2 Cellulose	No serious AE observed	<0.05
	(extract N/A) two 1340 ug hypericin -Tablet	(2 cycles)			Anxiety 41.15 ± 9.74 Crying 20.52 ± 11.73 Depression 29.26 ± 7.49 Craving 22.01 ± 11.03 Hydration 36.13 ± 8.50	Anxiety 23.08 ± 14.78 (p = 0.223) Crying 5.87 ± 10.23 (p = 0.001, 71% reduction) Depression 13.82 ± 6.48 (p < 0.001, 52% reduction) Craving 17.26 ± 7.41 (p < 0.001) Hydration 26.10 ± 10.18 (p < 0.090)	Tablets		
							(85)		
17	Xiao Yao San or Dan Zhi Xiao Yao San^8^ -Powder form	3/day	31	Confirmed	Diagnosed as PMS	Physical MDQ 68.9% reduction	Placebo	No AE	<0.001
		@ LP			Physical MDQ psychological MDQ BDI ANX ANG PSS diagnosed as PMS (p < 0.005)	Psychological MDQ 74.8% reduction	(30)		
		(3 cycles)							
						BDI 43.1% reduction ANX 23.8% reduction ANG 39.3% reduction PSS 16.4% reduction (p < 0.001)			
18	Crocus sativus (saffron) ^24^ 30 mg -Tablet	2/day	24	Confirmed	DSR < 50 PMS diagnosed by HDRS	50% reduction in severity of symptoms by DSR and HDRS (P < 0.001)	Placebo	No severe AE reported	<0.001
		(2 cycles)					(23)		
19	Elsholtzia splendens ^25^ 120 mg -Tablet	1/day	10	Not recorded	BDI 33.50 ± 5.82	BDI 23.60 ± 4.79 (p < 0.01) STAI 48.10 ± 5.20 (p < 0.05) STAI 52.00 ± 6.18	Placebo	None reported	<0.01
		(3 cycles)			STAI 58.40 ± 7.30		(10)		
					PAF 270.20 ± 82.61				
						PAF 176.7 ± 61.33 (p = 0.530)			
20	Cirsium japonicum^25^ 120 mg -Tablet	1/day	10	Not recorded	BDI 33.60 ± 8.8	BDI 30.40 ± 5.40	Placebo	None reported	<0.01
		(3 cycles)			STAI 50.90 ± 9.50	STAI 52.00 ± 6.18	(10)		
					PAF 257.30 ± 74.81	PAF 185.6 ± 53.65			
21	Ginkgo biloba L.^26^ 40 mg -Tablet	3/day	45	Confirmed	Overall score 34.80 (p = 0.930) Severity of psychological symptoms 38.41 (p = 0.899)	Overall score 11.11 (p < 0.001) Severity of psychological symptoms 10.89 (p < 0.001)	Placebo	No severe AE reported	<0.001
		@ LP					(45)		
		(2 cycles)							

Literatures yield 11 studies and 7 different herbs. It includes total dosage per day, number of times the herbs were taken per day dither at all phases or only during luteal phase (@ LP), the duration of the studies by menstrual cycles, sample size (Treatment Group: TG), two menstrual cycles of prospective ratings, baseline score using assessment tools used at each studies, the outcome measures and results, control types with number of analyzed: CG), and p-value.

*M, Measurement; PMSD, Premenstrual Syndrome Diary (four-point rating scale); PMTS, The Premenstrual Tension Syndrome Self-Rating Scale; DSR, Daily Symptom Report; PAF, Premenstrual Assessment Form;HDRS, Hamilton Depression Rating Scale (17-item); MD, Menstrual Diary(made up of 25 symptoms); MDQ, menstrual distress questionnaire); BDI, Beck Depression Inventory; ANX, state-anxiety; ANG, state-anger(ANX, ANG were measured with the Spielberger State Trait Personality Inventory; PSS, perceived stress scale; STAI.

**Study 10 [17] is the analysis of a sub-population of Study11 [18]. Study 12 is the analysis of a sub-population of a study within a systematic review of clinical trials [27].

### Overall symptoms

When comparing all the interventions reviewed in this study, hand moxibustion showed the highest rate of improval in overall assessment [11]. Notable improvements are as follows. Groups treated with Hand acupuncture, Vitex Agnus castus, and Xiao yao san have shown more than 70% improvement compared to their initial states [8,11,17-20]. For fatigue, Xiao yao san decoction resulted in a 68.9% improvement [8]. For insomnia, Xiao yao san decoction had a 74.8% improvement [8]. For avoidance of social activities and a desire to stay at home, hand moxibustion treatment showed more than 80% improvement in the treated group [11]. For the feeling of weight gain, hand moxibustion showed relief of the symptom [11]. For breast pain, Xiao yao san showed much improvement [8]. In cases of swelling, anxiety, mood swings, and depression, hand moxibustion showed the most improvement compared to other interventions [11]. For hot flashes, hand acupuncture showed more improvement than traditional acupuncture [11]. Improved symptoms resulting only from herbal medicinal interventions can be summarized as follows. For backache, Vitex Agnus castus showed more than a 50% improvement [19]. In swelling, St John’s wart showed the most improvement [21-23]. For anxiety, irritability, mood swings, depression, and tension, Xiao yao san showed the most improvement [8]. For increased anger during the luteal phase, Vitex Agnus castus and Elsholtzia splendens treatment resulted in more than a 50% improvement [17-20,25] (Table 4). Acupuncture treatment improved overall symptoms in all studies and all studies found AT to significantly outperform placebo [9-16] (Table 2). For the herbal interventions, all but Cirsium japonicum found a significant effect over placebo [8,17-26] (Table 3).

**Table 4:** Summary of improvements by symptoms

**Cluster of symptoms**		**Improved rate (%)**													
	**Interventions**	**AT**	**Hand Acu/Mox**		**Vitex Agnus castus**				**Hypericum perforatum**			**Xy**	**Es**	**Cj**	**Gb**
	**#**	**#1**	**#3**	**#4**	**#10**	**#11**	**#12**	**#13**	**#14**	**#15**	**#16**	**#17**	**#19**	**#20**	**#21**
	**Overall symptoms**	**54.9**	**80.9**	**83.5**	**50.1**	**78**	**50**	**47**	**51**	**69**	**43.5**	**71.9**	**29.5**	**9.5**	**68**
	**Physical symptoms**	**49.6**								**60.1**		**68.9**	**19**		**60.1**
	**Psychological symptoms**	**63.8**								**71.6**		**74.8**	**34.6**	**27.9**	**71.6**
Pain	Muscle stiffness	49.6					50					68.9	32.4		
	Headache				36.8			33	47.2						
	Cramps				38										
	Backache				51.3										
	Fatigue				59.8				35.5				33.6	35	
	General aches and pains				35.6										
Concentration	Insomnia	54.9			19.8				44.3			74.8			
	Forgetfulness								52.1						
	Confusion														
	Stay at home			83.3									49.7	41.8	
	Avoid social activities			100									37.9	23.8	
Autonomic reactions	Cold sweats	49.6													
	Hot flashes		95.7												
Water retention	Weight gain	49.6		97			50					68.9			
	Skin disorders														
	Painful breasts				63			58	21.9						
	Swelling			86.7	48.4				31.9	74.6	27.8		31.5	28.9	
Negative affect	Crying	54.9			41		50		71.2		71.3	74.8			
	Anxiety			92.3	58.8			38	39	65.7	43.9		44.3	29.7	
	Restlessness							42							
	Irritability				58.6				35				35.6	36.5	
	Mood swings		78.9	91.6	59.2				49				31.8	34.6	
	Depression			89	52.7			52	52.8				42.9	27.7	
	Tension														
Other symptoms	Change in eating habits				45		50		43.6	77.6	21.6				
	Abdominal bulging	49.6	74.3	78.2	55			60							
	Abdominal pain		76.2	81.9											
	Sensitivity to cold														
	Anger			100	59.1								50.5	36.9	

Treatment methods are numbered according to Tables 2 and 4. Improvements are recalculated using the following valuation: Significant result (%) = (baseline score-post treatment score)/baseline score) x100. The results were presented to reflect the results for symptom clusters.

AT: Acupuncture Treatment; Xy:Xiao Yao San; Es:Elsholtzia splendens; Cj:Cirsium japonicum; Gb:Ginkgo biloba L.; #: number according to Tables 2 and 3.

### Physical symptoms

Specific symptoms were examined in each intervention. In traditional acupuncture interventions, physical symptoms such as headache, cramps, backache, cold sweats, hot flashes, breast pain, skin disorders, swelling of hands and feet, sensitivity to cold, abdominal pain and bulging improved as much as 49.6% [9]. When specific items were examined in hand acupuncture intervention, abdominal pain and bloating were significantly reduced and hot flashes were significantly reduced [11] (Table 4).

### Psychological symptoms

With regard to psychological distress symptoms, rapid mood swings were significantly reduced [11]. In hand moxibustion treatments, abdominal pain and bloating were significantly reduced [11]. Water retention symptoms such as a sensation of weight gain and the swelling of hands or feet were significantly reduced [11]. Various psychological distress symptoms such as rapid mood swings, anger, impatience, depression, a desire to be alone, and lowered desires to talk or move were significantly reduced [11]. In one of the studies on an intervention with Vitex Agnus castus, headache, nervousness, restlessness, depression, breast pain and swelling, swelling and tympani have shown improvements over the control group [17]. In one of the studies on an intervention using Hypericum perforatum, the biggest improvements in score occurred for craving (77.6%) and hydration (74.6%) [21]. Depression and anxiety have also shown much improvement, while another study showed the biggest improvements in score for crying (71%) and depression (52%). Depression, craving, and hydration also had better results than the control group [23]. In Xiao Yao San, physical and psychological symptoms had been significantly reduced in that physical MDQ had 68.9% reduction in the treatment group compared to 18.6% reduction in placebo group and psychological MDQ had 74.8% reduction in the treatment group compared to 20.7% reduction in placebo group. [8]. In Elsholtzia splendens, the biggest improvements in score occurred for anger [25]. In Gingko biloba L., both psychological and physical symptoms had shown significant reduction [26] (Table 4).

### Risk of bias within studies

In a cross examination of comparing the rate of improval, variations of the assessment tools and different types of scales may have resulted in differences in the degree of improvements. Also, the detailed outcome of some studies were not included resulting in a possible risk of bias within studies. Re-evaluation of symptoms done is non-significant and the risk of bias in assessment appears because not all studies had the same reported symptoms of PMS/PMDD and the degree of symptoms vary between trials.

### Results of individual studies

The study on Korean hand acupuncture and moxibustion [11] has a significantly better outcome than the rest of the other studies on acupuncture intervention, which raises a question on the risk of bias within the study. Although the participants were randomly recruited by the advertisement placed on the university hospital board, since all the participants were nurses and since it was not a double nor single blinded study, there is a risk of information having been shared amongst the participants.

## Discussion

Alternative medicine has been widely used in the treatment of premenstrual syndrome. However, there has been limited evidence supporting both acupuncture and herbal medicine. Thus by reviewing randomized controlled trials of acupuncture and herbal medicine, this study aimed to identify the effectiveness of the alternative interventions.

In screening the RCTs, eight studies in acupuncture and 11 studies in herbal medicine that matched the criteria have been identified. Different acupuncture techniques such as traditional acupuncture, hand acupuncture and moxibustion, and traditional acupuncture technique with auricular points, have been selected [9-16]. In herbal medicine, studies on Vitex Agnus castus, Hypericum perforatum, Xiao yao san, Elsholtzia splendens, Cirsium japonicum, and Ginkgo biloba L. have been identified [8,17-26].

Our review aimed to review the acupuncture and herbal medical treatments for PMS/PMDD. The study found a favorable effect of acupuncture, moxibustion, herbal medicine over various controls. In the outcome of the acupuncture interventions, five studies showed an outcome that was better than the control group [12-16], and four studies showed more than a 50% reduction when compared to the initial state [9-11] (Table 2). In the outcome of the herbal interventions, all studies had a 50% or better improvement over control groups [8,17-26] (Table 3). The results of this study provide further support for previous evidence of the effectiveness of acupuncture shown in the systematic review done in 2011 by Kim *et al*. [6] as well as for studies on Vitex Agnus castus, Hypericum perforatum, Elsholtzia splendens, and Ginkgo biloba L. As for the study on hand acupuncture and moxibustion, it stated far better results than the rest of the other studies. Symptoms such as wanting to stay at home and anger diminishing in all women who complained of them at the baseline, resulted in a 100% improvement thus further investigation is need to identify any possible bias [11]. Also, no other previous evidence supports the result, thus more studies need to be conducted to support the current outcome. Furthermore, there were case studies that showed improvements on PMS/PMDD, however, due to the characteristic of this study, they were also excluded. On all acupuncture interventions, the outcome showed improvements better than the control groups thus our findings were consistent with case studies examining herbal interventions and acupuncture [28,29]. In a study done in Vitex Agnus castus with Fluoxetine as control, there was no significant differ rence between the two groups after the treatments [19]. According to Wood et al. [30], 20 mg doses per day of Fluoxetine reduced behavioral symptoms in 75% of cases and physical symptoms in 40%. A study done by Diegoli et al. [31] also observed that 20 mg of Fluoxetine per day had the remission rate of 65.4% which was the best rate when compared with other drugs such as Pyridoxine, Alprazolam, and Propranolol. According to the Diegoli et al. [31], Fluoxetine was more effective for treating isolation, confusion, crying, depression, weight loss, and emotional instability. Thus equivalence to Fluoxetine is actually a positive finding. The mechanism of acupuncture is possibly related to the regulative effects of acupuncture on the hormones-mediating receptors. In a double-blinded placebo-controlled animal study done on mice, with an acupuncture group and medication group modeled using Diethylstilbestrol and Ocytocin, the latency period between stretches was measured and vasopressin receptor in the uterus tissue was detected with reverse transcription polymerase chain reaction (RT-PCR) method. The stretch latent, stretch test to induce pain, was followed by acupuncture or two aforementioned medication resulting in the increased latency between “stretches” meaning its feeling less pain. According to the study, longer latency and less stretches resulted for the acupuncture group and a significant difference for the Ocytocin and vasopressin receptors in the control group [32]. Premenstrual syndrome is usually caused by Liver depression and Qi stagnation leading to transformation of Fire and disturbing cardiac sprit; or by invasion of Liver Qi into the Spleen and Stomach. PMS/PMDD is mainly caused by dysfunction of the Liver. According to the clinical symptoms, this syndrome pertains to the conceptions of headache, fever, body pain, edema, diarrhea, dizziness, abnormal emotional changes, distending pain in breasts before menstruation [33]. Herbal treatment aims to restore the dysfunction of Liver depression and Qi stagnation by readjusting the balance of the body [8]. Our findings were consistent with those of comparable reviews of acupuncture and herbal interventions for treating PMS/PMDD [34,35]. On a review of remedies used for PMS based on a national probability sample, those who have used acupuncture to relieve PMS pain, those who tried acupuncture found them effective [34]. Also, in the review on the efficacy and safety of specific herbal medications, phytomedicine such as Vitex Agnus castus(VAC) and dong gui, base remedy for Xiao yao san [8], have been proven of some efficacy for PMS when used in multiple-herb formulas [35]. Vitex Agnus castus (VAC) chaste tree has been proven in animal and clinical trials of its dopaminergic effects and its efficacy on PMS the most of those that has been investigated [18]. Hypericum perforatum influences the serotonergic system and suppresses proinflammatory cytokine levels [36]. It demonstrates to be an effective treatment for depression which is one of the symptoms of mood-related PMS symptoms [21]. Duvan et al., investigated oxidant/antioxidant status in PMS and found that increased oxidative stress and reduced anti-oxidant capacity may occur in PMS and an imbalance of oxidant/antioxidant systems may be a cause or the consequence of the various stress symptoms in PMS [37]. Elzholtzia splendens contains volatile oil and flavonoids and studies have reported that it had effects on reducing inflammation and fever [38]. According to Zou Y et al., the antioxidant mechanism of Hypericum perforatum attributed to its free radical scavenging activity, metal-chelation activity and reactive oxygen quenching activity that may lead to reducing PMS symptoms [39]. According to McKenna DJ, the antioxidant property of Ginkgo biloba leads to its exhibition of therapeutic activity in congestive symptoms of premenstrual syndrome [40]. Ginkgo biloba L. is rich in flavonoid glycoside and terpene lactone [26] and a published placebo-controlled trial on the efficacy of Ginkgo for the treatment of PMS was effective against the congestive symptoms of PMD [41]. Bioflavonoid, an active ingredient of Ginkgo, is known as a stress modulator which explains the usage of Ginkgo as an anxiolytic medicine for PMS [26]. Hence, treatment targeting these mechanisms may exert their benefits in PMS/PMDD by correcting underlying dysfunctions. As for the quality of the study, on the review of acupuncture treatments, the sample sizes varied ranging from 7 to 35, and 17 to 101 on herbal treatments. Although the sizes of the trials differ greatly, all included studies except for the study on Xiao Yao San met the Jadad scale criteria on herbal treatment [42]. This review shows a wide spectrum of traditional treatment methods which is not limited to one method of CAM, but acupuncture and herbal medicine combined, thus giving a better idea of what to expect in treating PMS/PMDD with traditional medicine. Also, by examining the best treatment methods for specific symptoms by reviewing the rate of improval categorized by symptoms, this review may be used as a guideline in treatment method selection for the different occurring symptoms personalized to each PMS/PMDD patient.

### Limitations

Although acupuncture and herbal medicine are largely practiced amongst Eastern medical doctors, there were limited RCTs available in this research. We have tried to evaluate the effective acupuncture and herbal medical treatment methods for PMS/PMDD by reviewing RCTs. Although the risk of bias was evaluated for all included trials that were reviewed, lack of quality of trials with reference to any particular method may limit the quality of the review. Many of the researches were done one time only for each intervention which makes it difficult to state that they were significantly meaningful. On the contrary, on the effectiveness of Vitex Agnus castus performances respect with placebo has been identified and under research for at least 4 different trials. However, the review lacks the quality evaluation of trials included in the review on Vitex Agnus castus. The research done had different inclusion criteria, measuring methods, degree of symptom severity, sample sizes, diagnoses, and treatment sessions. Only limited number of studies had two cycles of prospective ratings recorded prior to study entry for acupuncture intervention. Also, much research has been conducted on phototherapy prior to these inclusion dates. Those researches conducted within the inclusion dates that are not included in this review have been excluded at the electronic database search stage. A study done by Kilicdag EB on Fructus agni casti and bromocriptine for treatment of hyperprolactinemia and mastalgia published in 2004 has also been excluded due to the above reason.

## Conclusions

Eight acupuncture treatments and 11 herbal medical treatments were identified and evaluated. Acupuncture treatments included general acupuncture points, manipulated techniques of acupuncture, and hand acupuncture. Herbal medical treatments included the following formulae: Xiao yao san (or Dan Zhi Xiao yao san) and included herbal medicine are Vitex Agnus castus, Hypericum perforatum, Crocus sativus, Elsholtzia splendens, Cirsium japonicum, and Ginkgo biloba L. The data presented here provide support for the effectiveness of acupuncture and herbal medicine in premenstrual syndrome and premenstrual dysphoric disorder with a 50% or better reduction in symptoms than the initial state. Overall, in acupuncture treatment, it can be concluded that the safety of the treatment has been proven by no report of major adverse events, treatment sessions as few as 2 ~ 4 sessions show a 77.8% reduction in the symptoms and since more treatments of up to 30 sessions does not increase the degree of symptom relief, frequency of the treatment does not affect the outcome result. Also, there was no difference between luteal phase and follicular phases in the treatment result thus the sessions need not be limited only to the luteal phase. In herbal treatment, there have been no serious adverse events reported which in turn proves its safety with the recommended dosage. The majority of studies lasted 2 to 3 menstrual cycles and resulted in the relief of PMS/PMDD symptoms. However further investigation is needed on the maintenance of symptom relief. Even though much study has been done prior to these inclusion dates as mentioned above, large-scale, multicenter randomized, double-blind and placebo-controlled clinical researches are needed to support the results since most interventions had only one study conducted on them. In further research, comparison between the frequency, dosage, and treatment duration for each intervention on each PMD/PMDD symptom may lead to much specific guidance in a clinical setting.

## Competing interests

The authors declare that they have no competing interests.

## Authors’ contributions

SHJ contributed to the collection and overall analysis of studies. DIK contributed to the collection, analysis, and organization of the initial draft. DIK and MSC conducted a critical review of the manuscript and provided final editing to the manuscript. All authors read and approved the final manuscript.

## Pre-publication history

The pre-publication history for this paper can be accessed here:

http://www.biomedcentral.com/1472-6882/14/11/prepub

## Supplementary Material

Additional file 1 PRISMA 2009 checklist. Click here for file
